# Severe Adenoviral Pneumonia in an Immunocompetent Host with Persistent Fevers Treated with Multiple Empiric Antibiotics for Presumed Bacterial Co-Infection: An Antibiotic Stewardship Perspective on De-Escalation Derailed

**DOI:** 10.3390/jcm6040042

**Published:** 2017-04-04

**Authors:** Burke A. Cunha, John Gian, Natalie C. Klein

**Affiliations:** 1 Infectious Disease Division, Winthrop-University Hospital, 222 Station Plaza North (Suite #432), Mineola, New York, NY 11501, USA; jgian@winthrop.org (J.G.); nklein@winthrop.org (N.C.K.); 2 School of Medicine, State University of New York, Stony Brook, New York, NY 11794, USA

**Keywords:** viral pneumonia, conjunctival suffusion, clinical significance of fever, benefits of fever, empiric antibiotic therapy, antibiotic de-escalation, viral and bacterial co-infection

## Abstract

We present a case of severe adenoviral pneumonia in a 20-year-old immunocompetent host with persistently high fevers. The patient was needlessly given multiple empiric antibiotics for non-existent bacterial co-infection. This case has important antibiotic stewardship lessons for practitioners in approaching fevers in the ICU.

## 1. Introduction

Traditionally, adenoviral pneumonia occurs in children and military recruits, but recently has been recognized as an important cause of viral pneumonia in non-military immunocompetent adults [1]. Adenoviral community acquired pneumonia (CAP) is one of the few causes of viral pneumonia with focal infiltrates on chest X-ray (CXR) that may mimic typical bacterial CAPs or atypical CAPs, (e.g., Legionnaire’s disease) [2]. There are no well-documented reports of bacterial co-infection in hospitalized adults with adenoviral pneumonia in normal hosts [3]. Because the CXR appearance of adenoviral CAP may resemble bacterial CAP, clinicians must be careful not to ascribe focal infiltrates of adenoviral pneumonia to superimposed bacterial co-infection. 

We report a case of severe adenoviral CAP in an immunocompetent host excessively treated empirically with multiple antibiotics for persistent fevers, presumably due to bacterial co-infection.

## 2. Case

Twenty-year-old male presented with an influenza-like illness (LI) with fever, cough, wheezing, shortness of breath, and myalgias. His symptoms began 3 days prior to hospitalization, and he visited an Urgent Care Center and was started on amoxicillin. On admission, his temperature was 38 °C (101.8 °F), pulse was 125/min, and respiratory rate was 22/min. Physical exam was unremarkable except for wheezing and conjunctival suffusion. His white blood cell (WBC) count was 5.7 K/mm^3^ (81% PMNs, 8% lymphocytes, 11% monocytes) and platelet count was 158 K/µL (*n* > 160 K/mm^3^). Serum transaminases and alkaline phosphatase were normal. His C-reactive protein (CRP) was 110 mg/L (*n* < 3 mg/L) and procalcitonin (PCT) was 0.12 mg/mL (*n* < 0.5 mg/mL). CXR showed a right middle lobe infiltrate thought to represent a bacterial CAP, and the patient was empirically started on ceftriaxone. Respiratory viral PCR (Film Array) was positive for adenovirus. Moxifloxacin was started on hospital day (HD) #3. He was transferred to the medical intensive care unit (ICU), and his temperature was 102.6 °F, pulse was 116/min, respiratory rate was 30/min, and oxygen saturation was 92% on oxygen at 4 L/min. Repeat CXR showed worsening pneumonia, involving right middle and lower lobes. Multiple sputum and blood cultures—obtained prior to antibiotic therapy—were negative. Empiric antibiotics were changed to meropenem, vancomycin, and levofloxacin for presumed bacterial co-infection.

**Figure 1:**
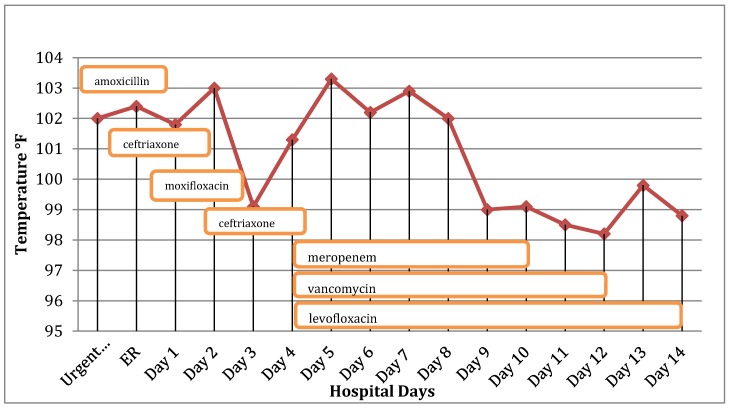
Temperature (°F) and antibiotics prescribed over time.

On HD #4, fevers continued, and he was intubated. WBC count remained normal at 6.2 K/mm^3^. His erythrocyte sedimentation rate (ESR) was 35 mm/h, CRP was 92 mg/L, and his PCT was 0.08 mg/mL (*n* < 0.5 mg/mL). Cold agglutinin titer was negative. *Legionella sp.* antibody and urine antigen were negative. Sputum and nasopharyngeal viral cultures were both positive for adenovirus. Repeat respiratory PCR remained positive for adenovirus, and his adenovirus titer was elevated at 1:128 (*n* ≤ 1:8). A dose of cidofovir was given. Since the fevers were clearly due to adenoviral pneumonia, multiple empiric antibiotics had no effect on his fevers. Accordingly, the infectious disease consultant repeatedly recommended discontinuing antibiotics for “presumed bacterial coinfection”. However, meropenem, levofloxacin, and vancomycin were continued for “possible bacterial co-infection” because the patient remained febrile (Figure 1). 

He was extubated on HD #8, but his 102 °F fevers continued. Repeat PCT was again unremarkable at 0.17 mg/mL (<0.5 mg/mL). CXR showed much improved right lung infiltrates, and by HD #12 he became afebrile and his CXR infiltrates had resolved. The eventual resolution of his severe adenoviral CAP should not be ascribed to a single dose of cidofovir.

## 3. Discussion

Adenovirus is an uncommon cause of CAP (2%) in non-military immunocompetent hospitalized adults, and is responsible for 15% of severe CAP [1,3]. 

The patient was a young immunocompetent adult that was persistently febrile, but his WBC count was normal, with mild thrombocytopenia which argued against bacterial co-infection. Sputum and blood cultures were negative. *Legionella* sp. titer and urine antigen were negative. Viral PCR did not detect influenza or other influenza-like illnesses (ILI) respiratory viruses in his respiratory secretions. Excluding influenza and adenoviral pneumonia, no other cause of bacterial CAP is associated with conjunctival suffusion. PCR was repeatedly positive for adenovirus. Sputum viral culture and shell vial assay were positive for adenovirus, and his adenoviral titer was highly elevated at 1:128 (*n* < 1:8). 

Antimicrobial stewardship programs (ASP) have many positive effects. Unnecessary antibiotics impact cost, length of stay, risk of adverse events, and potential antimicrobial resistance [4,5]. Multiple antibiotics with no effect on his fevers were still administered in spite of a proven diagnosis of adenoviral pneumonia. Fever is an important host defense mechanism, and is also an indicator of infection resolution [6,7]. Unfortunately, the benefits of fever (i.e., decreased viral replication) are often overlooked. All too often, fever signifies ongoing bacterial infection or newly-acquired antibiotic resistance [6,7,8,9,10]. Admitted immunocompetent adults with viral CAPs (e.g., influenza, adenovirus) are expectedly febrile until fever-mediated host defenses resolve the viral pneumonia.

Because of the well-known association of influenza pneumonia with bacterial co-infection, many physicians assume that bacterial co-infections are common with other viral pneumonias [2]. Bacterial co-infection in ILI viral pneumonias (e.g., RSV, hMPV, HPIV 1–4) are reportedly rare. In hospitalized adults with adenoviral CAP, there are no well-documented reports of bacterial co-infection [1,3,8]. This case illustrates this important point well. Although there was nothing clinically to suggest bacterial co-infection, it was assumed there was a superimposed bacterial co-infection only because of persistent fevers [2,8,9,10].

As improbable as this assumption was, it did not stand to reason, since the fever from the presumed bacterial co-infection did not decrease with multiple antibiotics. In spite of clinical probability and repeated infectious disease recommendations, multiple antibiotics were continued for days. In this case, there were fortunately no antibiotic adverse effects, but unnecessary antibiotic costs were incurred. Furthermore, indiscriminate antibiotic use may foster antibiotic resistance [2,9,10]. 

Therefore, from an ASP perspective, this “real-world” case demonstrates several important clinical lessons. Firstly, unless it is a threat to the host, fever is good and not bad [10]. Secondly, fevers do not indicate bacterial infection, per se, and fevers due to bacterial infection rapidly decrease with appropriate therapy. Unfortunately, in this case, “no potential bacterial pathogen was left untreated”. Thirdly, empiric antibiotic therapy should be based on clinical probability, and rampant empiricism should be avoided. Early empiric therapy with an appropriate antibiotic before diagnostic test results are reported is reasonable. However, after the diagnosis of adenoviral CAP was confirmed, multiple antibiotics were continued without basis to treat a presumed bacterial co-infection. If any bacterial co-infection was present, any one of the several antibiotics given would have rapidly decreased the fevers. From an ASP standpoint, with multiple empiric antibiotics, less is more. This patient needed ventilatory and circulatory support until his adenoviral pneumonia resolved. Once again, intensive care, not intensive antibiotics, was responsible for his survival [8].
